# Research Productivity of Canadian Radiation Oncology Residents: A Time-Trend Analysis

**DOI:** 10.3390/curroncol28010003

**Published:** 2020-11-30

**Authors:** Adam Mutsaers, Sangyang Jia, Andrew Warner, Timothy K. Nguyen, Joanna M. Laba, David A. Palma

**Affiliations:** 1 Department of Radiation Oncology, London Health Sciences Centre, London, ON N6A 5W9, Canada; adam.mutsaers@lhsc.on.ca (A.M.); andrew.warner@lhsc.on.ca (A.W.); timothy.nguyen@lhsc.on.ca (T.K.N.); joanna.laba@lhsc.on.ca (J.M.L.); 2 Schulich School of Medicine & Dentistry, Western University, London, ON N6A 3K7, Canada; sjia2020@meds.uwo.ca

**Keywords:** resident research, academic productivity, publications, radiation oncology

## Abstract

(1) Background: Research productivity is a mandatory component of Canadian radiation oncology (RO) resident training. To our knowledge, Canadian RO resident research publication productivity has not previously been analysed. (2) Methods: We compiled a 12-year database of RO residents in Canadian training programs who completed residency between June 2005 and June 2016. Resident names and dates of training were abstracted from provincial databases and department websites and were used to abstract data from PubMed, including training program, publication year, journal, type of research, topic and authorship position. Residents were divided into four time periods and the linear trend test evaluated publication rates over time. Univariable and multivariable logistic regression analyses were performed to identify authorship predictors. (3) Results: 227 RO residents representing 363 publications were identified. The majority were first-author publications (56%) and original research (77%). Overall, 82% of first-author, and 80% of any-author articles were published in resident year 4 or higher. Mean number of publications for first-author and any-author positions increased significantly over time (*p* = 0.016 and *p* = 0.039, respectively). After adjusting for gender and time period, large institutions (> 3 residents per year) trended toward associations with more first-author publications (odds ratio (OR): 2.44; *p* = 0.066) and more any-author publications (OR: 2.49; *p* = 0.052). No significant differences were observed by gender. (4) Conclusions: Canadian RO resident publication productivity nearly doubled over a 12-year period. The majority of publications are released in the last 2 years of residency, and larger residency programs may be associated with more publications. These findings serve as a baseline as programs transition to Competency Based Medical Education (CBME).

## 1. Introduction

Scholarly pursuits during medical residency training have been a core part of the CanMEDs framework since its introduction in Canada in 1996 [1]. Academic research is a required component of radiation oncology (RO) residency training programs. Specifically, to complete subspecialty certification in Canada, the Royal College of Physicians and Surgeons of Canada requires completion of a scholarly project warranting academic publication or national conference presentation during residency training [2]. In the recently introduced Competency Based Medical Education (CBME) curriculum, the execution of a scholarly project relevant to the specialty is an entrustable professional activity (EPA) required for completion of the program. In many programs, productivity is encouraged beyond this minimum as a valuable learning experience, to provide contribution to the field of RO, and as a service component to training. Developing competency in research during residency may help residents critically interpret evidence in practice and continue to contribute to the field as fellows and staff.

While American RO resident productivity has been analysed, [3,4] the productivity of RO residents in Canada has not been previously studied. The purpose of this study was to identify changes in the quantity of Canadian trained RO resident publications over time, and to examine potential drivers of resident academic productivity. This study can provide a reference point for interested medical students, active RO residents and their programs, and for potential employers.

## 2. Methods

### 2.1. Data Collection

We compiled a 12-year database of RO residents in Canadian training programs who completed residency between June 2005 and June 2016. Resident names and dates of training were abstracted from provincial physician databases (e.g., College of Physicians and Surgeons of Ontario), hospital and department websites, and online professional sites. Productivity was measured by the number of PubMed-indexed publications during residency and 6 months thereafter, in an attempt to include work completed primarily in residency. Abstracted data included training program, year of publication, publication journal, type of research, topic of research and authorship position. Journal impact factors were obtained from Scimago Journal and Country Rank (www.scimagojr.com), by year of publication.

### 2.2. Statistical Analysis

Residents were divided into 4 cohorts based on 3-year time periods, representing graduates from: 2005–2007 (*n* = 41), 2008–2010 (*n* = 62), 2011–2013 (*n* = 65), and 2014–2016 (*n* = 59). Descriptive statistics were generated for all residents and stratified by first-author publication (yes versus no) and any-author publication (yes versus no), compared using the chi-square test, Fisher’s exact test or Wilcoxon rank sum test as appropriate. Linear trend test was used to evaluate changes in publication rates over time based on the pre-defined time period cohorts.

Univariable and multivariable logistic regression were performed to identify significant predictors of first-author and any-author publication based on the following characteristics: program (univariable only), gender, resident year (defined as postgraduate year (PGY) on publication date), time period, and size of program (small, medium or large; defined as number of residents in program over the last 12 years). Institutions and programs were anonymised for reporting. All statistical analyses were performed using SAS version 9.4 software (SAS Institute, Cary, NC, USA) using two-sided statistical testing at the 0.05 significance level.

## 3. Results

### 3.1. Publication Characteristics

**Figure 1:**
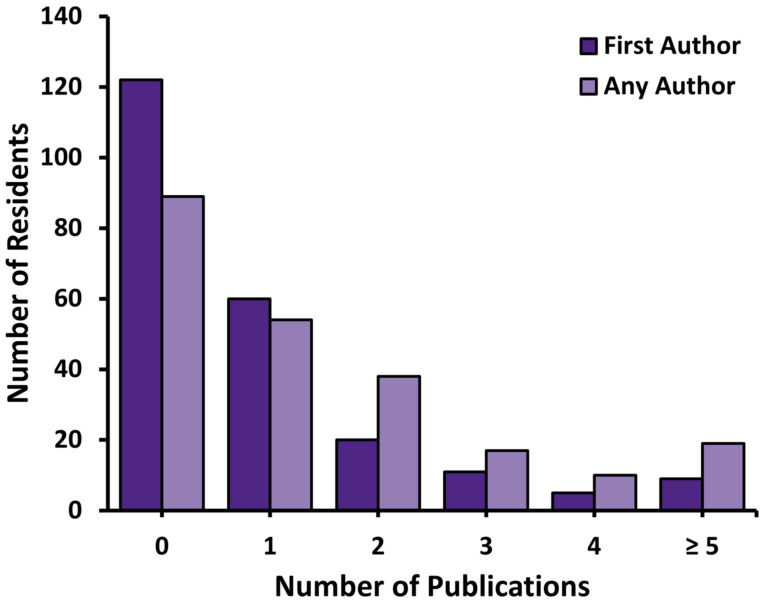
Number of publications per resident across all time periods.

A total of 227 RO residents graduating from 13 Canadian RO residency programs between 2005 and 2016 were identified, collectively co-authoring 363 publications across 116 journals (Table 1; Supplemental Tables S1 and S2). The distributions of first and total author publications are shown in Figure 1. The majority (56%, *n* = 205) were first-author publications, with 18% as second authorships, and 26% representing later author publications. Eighty-four residents (37%: 95% confidence interval (CI): 31–43%) co-authored at least 2 publications, with 45 of those (20% CI; 95% CI: 15–25%) publishing at least twice as first-author. Nearly half of papers (42%, *n* = 153) were published in journals with an impact factor greater than or equal to 4.0 at the time of their publication. The most common journals (Table 2) were International Journal of Radiation Oncology * Biology * Physics (20%, *n* = 72) and Radiotherapy & Oncology (7%, *n* = 26).

**Figure 2:**
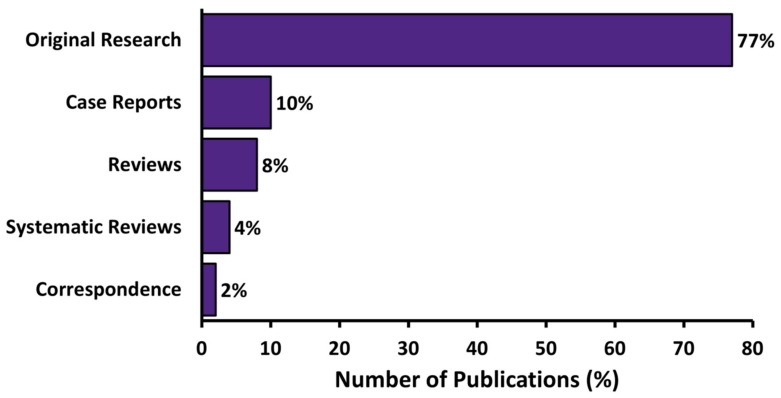
Percentage of publications by research category.

Most articles were original research (77%, *n* = 280), while case reports, reviews, systematic reviews and correspondence represented 10%, 8%, 4% and 2%, respectively (Figure 2). Thirty-nine percent of residents had no publications to report during training. The majority of publications were authored by senior residents (defined as PGY-4 or above) representing 82% of first-author, and 80% of any-author articles.

### 3.2. Time Trend Analysis

**Figure 3:**
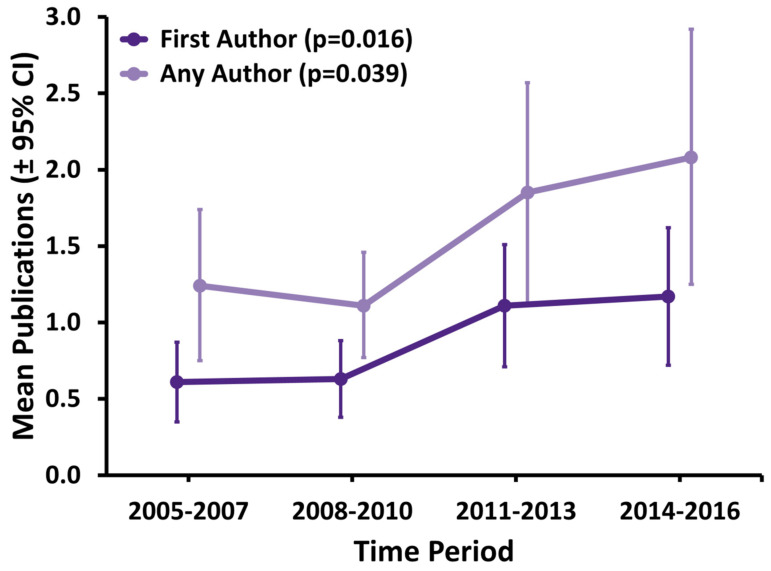
Summary of publication rates for first- and any-author publications by time period (*n* = 227). *p*-values reported from linear trend test. Abbreviations: CI = confidence interval.

The results from the time trend analysis are shown in Figure 3. The mean number of first-author publications per resident increased significantly over time from 0.61 (95% CI: 0.35–0.87) in 2005–2007 to 1.17 (0.72–1.62; linear trend test *p* = 0.016) in 2014–2016. Similarly, total publications (with any-author position) increased from 1.24 (0.75–1.74) to 2.08 (1.25–2.92; *p* = 0.039). This corresponded with increases in the maximum observed number of first-authored and total publications starting with 2 and 7 in 2005–2007 and increasing to 8 and 21 in 2014–2016, respectively.

### 3.3. First-Author Publications

On univariable logistic regression analysis for predictors of first-author publications shown in Table 3, one of the 13 institutions (labelled institution A) was associated with more first-author publications than the others (OR: 2.24; 95% CI: 1.19–4.22; *p* = 0.013). Another (institution B) was associated with fewer first-authored publications than all others (OR: 0.27; 95% CI: 0.07–0.98; *p* = 0.047). A trend towards significance was noted comparing large versus small institutions (OR: 2.45; 95% CI: 0.95–6.30; *p* = 0.063), but this was less predictive overall when including all institution sizes (*p* = 0.135). Neither gender (*p* = 0.410) nor time period (*p* = 0.365) were predictive. Multivariable analysis did not identify any significant predictors of more first-author publications, however large vs. small institutions predicted for more first-author publications (OR: 2.44; 95% CI: 0.94–6.33; *p* = 0.066).

### 3.4. Any-Author Publications

For authorship at any position, large versus small institutions was significantly predictive (OR: 2.55; 95% CI: 1.02–6.33; *p* = 0.044), although this was not significant when including medium institutions (*p* = 0.130) (Supplemental Table S2). One institution (labelled institution C) was found to be associated with more publications for any-authorship (OR: 8.38; 95% CI: 1.07–65.63; *p* = 0.043). Correspondingly, neither gender (*p* = 0.371) nor time period (*p* = 0.351) were predictive. Similarly, multivariable analysis did not identify any significant predictors of more any-author publications. Large vs. small institutions (OR: 2.49; 95% CI: 0.99–6.24; *p* = 0.052) and 2011–2013 compared to 2005–2007 (OR: 1.89; 95% CI: 0.84–4.28; *p* = 0.127) non-significantly predicted for more any-author publications.

## 4. Discussion

Canadian-trained RO resident publications have significantly increased over the past 12 years. Mean first-author publications per resident during residency increased from 0.61 (95% CI: 0.35–0.87) in 2005–2007 to 1.17 (0.72–1.62; linear trend test *p* = 0.016) in 2014–2016, while mean any-author publications increased from 1.24 (0.75–1.74) to 2.08 (1.25–2.92; *p* = 0.039). The majority of publications were authored by residents in PGY-4 or higher. These findings provide a benchmark for medical students interested in pursuing a career in RO, for RO residents in training, for program and research directors, for potential employers and can serve as a baseline for CBME implementation.

This significant increase in publications over time could have several underlying causes. Firstly, changing demand and competitiveness in Canadian RO employment availability could have promoted increases in productivity as applicants attempted to position themselves for jobs or fellowships [5]. Secondly, academic inflation is a well-described trend in academia in general and in RO specifically. Ojerholm et al. documented a dramatic increase in number of authors per publication in major RO journals over a thirty-year period. Additionally, the number of trainee first-author publications grew from 16% of publications to 56% between 1984 and 2014 [6]. Thirdly, residency programs may be increasing focus, training, and resources toward resident research productivity. Fourthly, the increase in resident productivity naturally creates more experience within training programs and mentors, potentially giving rise to a virtuous cycle of mentorship and publication in each new cohort of residents. Finally, changing resident demographics, including more trainees with advanced or research focussed degrees (not captured in study) may also be contributory.

The rate of non-publishing residents remained stable over the course of our analysis (mean: 39%, range: 31–44%), suggesting that the increase in publications detected herein is most attributable to a near doubling of publications among publishing residents, rather than an increase in the percentage of residents who publish. This implies some inertia with non-publishing residents that has yet to be overcome despite the overall trend in increasing publications. This may represent a population of focus for program or research directors going forward.

Two institutions were associated with a significantly higher likelihood of publication (first-author publication for institution A and any publication for institution C) whereas one institution (institution B) was associated with a significantly lower likelihood of first-author publication, suggesting that local factors may play a role in resident productivity. Residents at large institutions (compared to small) may also be more likely to publish. Institutional support systems, library and data analysis supports, staff research mentors, clinical workload for residents, and cultural factors may all be drivers.

First-author productivity in United States RO residency programs was studied by Morgan et al. for the years 2002–2007, and by Verma et al. for 2014–2015 [3,7]. A similar trend in increasing publication rates was noted between 2007 and 2014 [3]. However, when compared with rates of publication over similar time periods, our results suggest Canadian RO residents have published less, despite using more lenient publication date restrictions in our search. Canadian resident first-author mean publication rates were lower than US counterparts (mean first-author publications in 2005–2007 cohort: 0.61 vs. 1.01; 2014–2016 cohort: 1.17 vs. 2.00) in both analysed time periods. While reasons for this difference are not immediately evident, they may warrant further investigation.

Overall, limited data exist on academic productivity of Canadian resident physicians. Canadian otolaryngology residents published a mean of 3.35 any-author publications during residency training between 1998 and 2013 [8]. A self-reported survey of 42 Canadian urology residents across 10 programs in 2013 revealed a mean publication rate of 1.25 [9], while a similar survey of 85 orthopaedic residents showed a mean rate of 0.45 publications, though not all had completed training [10].

While the mandatory completion of an academic project during residency has existed as a Canadian RO Royal College requirement since at least 2012, the 2019 introduction of the CBME curriculum has changed the education landscape [11]. The requirement to “contribute to a research program” carries forward from prior requirements, but the assessed research competencies have expanded to include the clinically applicable skills of critical appraisal, research translation and presentation, and an understanding of the research process and ethics [12]. The curriculum and the academic project requirements within it are likely to continue to evolve and the impact on resident research output remains to be seen.

Debate remains about whether increased focus on scholarly productivity is beneficial to trainees. Resident productivity has been associated with increased departmental costs, and a reduced clinical caseload [13]. However, resident publication rates have also been associated with positive clinical evaluations of residents, raising the question of causality [14]. While some evidence suggests publications as a medical student can predict resident productivity, [8] correlation with fellowship and staff productivity remain less clear.

Should continued productivity increases be desired by programs or residents, there are several interventions that have shown benefits for resident productivity. Lack of designated time, mentorship, interest, funding and technical support have been identified as challenges in research productivity during residency [15]. Gutovich et al. conducted a survey of RO residents and new staff and identified dedicated, protected time in residency for research as the sole significant predictor of output [16]. In other specialities, resident productivity has increased with points-based reward systems, mandatory productivity minimums, simultaneous masters or PhD degree program completion, service hour restrictions, peer group workshops, structured research curriculum and rotations, access to statistical support and training, and staff mentorship with proven productivity have been found to improve journal publication rates [17,18,19,20,21,22,23,24,25,26,27,28].

## 5. Limitations

The findings of this study should be considered in the context of its limitations. While our estimated capture rate of RO trainees exceeds 90%, potential issues with data sufficiency and completeness may impact results. Resident demographic data were not available in a standard, centralised format. The degree of detail available in provincial physician databases was variable and thus the number of eligible residents in certain regions (Quebec, Maritimes) may be underrepresented. Furthermore, Canadian-trained ROs who are not currently employed in Canada were not easily identified. Valuable academic activities outside of PubMed-indexed publications (such as grant applications) were also not captured. Several studies correlated simultaneous or previous completion of advanced degrees with increased productivity in residency [19,20,21]. Unfortunately, this was unable to be analysed in our dataset. Following the date of graduation, a 6-month window was utilised in an attempt to capture publications related to work in residency, but published after. A more time-intensive methodology may have more accurately tied publications linked to residency. Finally, the analysis primarily measures quantity of publications and not the quality or magnitude of the work. Measures of quality like h-index were considered but were not explored because residents all had similarly low scores within a small range.

## 6. Future Directions

While studies have found resident productivity to be a mixed predictor of future publication rates, further work is required to determine the impact of resident publication rate on research productivity during later years, and to assess interventions to provide additional research mentorship and support if needed.

## 7. Conclusions

Canadian RO resident publication productivity nearly doubled over a 12-year period. Year of graduation was significantly related to both first-author and total publications during residency and larger institution size for total publications. A single institution was associated with higher rates of first-author publications, while another institution correlated with higher rates of any-author publications. Future directions include analysing productivity outside of publications, and exploring initiatives to facilitate academic output in centres or among residents that wish to do so.

## Figures and Tables

**Table 1:** Baseline characteristics of publishing residents (*n* = 227).

Characteristic	All Publications (*n* = 227)
**Gender**—*n* (%)	
Male	132 (58.2)
Female	95 (41.9)
**Cohort**—*n* (%)	
2005–2007	41 (18.1)
2008–2010	62 (27.3)
2011–2013	65 (28.6)
2014–2016	59 (26.0)
**Resident Year**—mean ± SD (95% CI)	2.85 ± 2.51 (2.52, 3.18)
**Institution Size**—*n* (%)	
Small	25 (11.0)
Medium	118 (52.0)
Large	84 (37.0)

CI = confidence interval.

**Table 2:** Summary of the 8 most common journals for any radiation oncology (RO)-resident authored publications (*n* = 363).

Journal	Publications *n* (%)	Impact Factor (2015)
Int. J. Radiat. Oncol. Biol. Phys.	72 (19.8%)	4.99
Radiother. Oncol.	25 (6.9%)	5.35
Can. Urol. Assoc. J.	18 (5.0%)	0.97
Support. Care Cancer	14 (3.9%)	2.84
Curr. Oncol.	13 (3.6%)	2.10
Radiat. Oncol.	13 (3.6%)	3.06
Brachytherapy	11 (3.0%)	3.01
Pract. Radiat. Oncol.	7 (1.9%)	1.87

**Table 3:** Univariable and multivariable logistic regression models predictive of first-author and any-author publications (*n* = 227).

Dependent Variable:	First-Author Publication		Any-Author Publication	
Variable:	OR (95% CI)	*p*-Value	OR (95% CI)	*p*-Value
**Univariable:**				
**Male vs. Female**	0.80 (0.47, 1.36)	0.410	0.78 (0.45, 1.34)	0.371
**Cohort (vs. 2005–2007)**		0.365		0.351
2008–2010	1.06 (0.47, 2.37)	0.895	1.12 (0.51, 2.47)	0.780
2011–2013	1.71 (0.78, 3.79)	0.184	1.94 (0.87, 4.36)	0.108
2014–2016	1.62 (0.72, 3.63)	0.245	1.35 (0.60, 3.03)	0.464
**Institution Size**		0.135		0.130
Medium vs. Small	1.67 (0.67, 4.18)	0.270	1.92 (0.80, 4.60)	0.142
Large vs. Small	2.45 (0.95, 6.30)	0.063	2.55 (1.02, 6.33)	**0.044**
**^a^ Institution (vs. Other)**		0.174		0.080
A	2.24 (1.19, 4.22)	**0.013**	1.61 (0.83, 3.12)	0.158
B	0.27 (0.07, 0.98)	**0.047**	0.54 (0.19, 1.55)	0.252
C	2.77 (0.83, 9.26)	0.099	8.38 (1.07, 65.63)	**0.043**
D	0.46 (0.16, 1.35)	0.156	0.42 (0.15, 1.15)	0.093
E	1.53 (0.68, 3.43)	0.304	1.33 (0.57, 3.12)	0.507
F	0.56 (0.19, 1.69)	0.305	0.40 (0.14, 1.18)	0.097
G	0.56 (0.17, 1.93)	0.362	0.63 (0.20, 2.02)	0.435
H	0.67 (0.28, 1.60)	0.365	0.51 (0.22, 1.19)	0.117
I	1.48 (0.39, 5.64)	0.570	2.32 (0.47, 11.45)	0.300
J	1.17 (0.29, 4.79)	0.828	1.08 (0.25, 4.62)	0.920
K	1.04 (0.39, 2.79)	0.945	3.24 (0.90, 11.60)	0.071
L	1.00 (0.32, 3.06)	0.994	1.48 (0.44, 4.97)	0.523
**Multivariable:**				
**Male vs. Female**	0.83 (0.49, 1.43)	0.511	0.82 (0.47, 1.42)	0.472
**Cohort (vs. 2005–2007)**		0.379		0.395
2008–2010	1.03 (0.46, 2.34)	0.940	1.11 (0.50, 2.47)	0.805
2011–2013	1.66 (0.75, 3.71)	0.214	1.89 (0.84, 4.28)	0.127
2014–2016	1.63 (0.72, 3.69)	0.241	1.35 (0.60, 3.06)	0.468
**Institution Size**		0.128		0.145
Medium vs. Small	1.61 (0.64, 4.05)	0.316	1.84 (0.76, 4.44)	0.175
Large vs. Small	2.44 (0.94, 6.33)	0.066	2.49 (0.99, 6.24)	0.052

OR = odds ratio, CI = confidence interval; *p*-values < 0.05 shown in **bold**; ^a^ not considered for multivariable modelling.

## Supplementary Materials

The following are available online at https://www.mdpi.com/1718-7729/28/1/3/s1, Table S1. Baseline characteristics of publishing residents stratified by first author publication reported per resident (*n* = 227). Table S2. Baseline characteristics of publishing residents stratified by any author publication reported per resident (*n* = 227).
