# Supplementary material for: Research Productivity of Canadian Radiation Oncology Residents: A Time-Trend Analysis
Source: Curr Oncol. 2020 Nov 30;28(1):4–12. doi: 10.3390/curroncol28010003 (PMC7816183; doi:10.3390/curroncol28010003)
Supplement: Supplementary file 1 [file curroncol-28-00003-s001.pdf]

Table S1. Baseline characteristics of publishing residents stratified by first author publication reported per resident (n=227).

Characteristic	Residents (n=227)	First Author Publication		
		Yes (n=105)	No (n=122)	p-value
**Gender** – n(%)				
Male	132 (58.2)	58 (43.9)	74 (56.1)	0.409
Female	95 (41.9)	47 (49.5)	48 (50.5)	
**Cohort** – n(%)				
2005-2007	41 (18.1)	16 (39.0)	25 (61.0)	0.362
2008-2010	62 (27.3)	25 (40.3)	37 (59.7)	
2011-2013	65 (28.6)	34 (52.3)	31 (47.7)	
2014-2016	59 (26.0)	30 (50.9)	29 (49.2)	
**Resident Year** – mean ± SD (95% CI)	2.85 ± 2.51 (2.52, 3.18)	4.69 ± 1.28 (4.44, 4.93)	1.27 ± 2.21 (0.88, 1.67)	**< 0.001**
**Institution Size** – n(%)				
Small	25 (11.0)	8 (32.0)	17 (68.0)	0.130
Medium	118 (52.0)	52 (44.1)	66 (55.9)	
Large	84 (37.0)	45 (53.6)	39 (46.4)	

CI = Confidence interval; P-values < 0.05 shown in **bold**.

Table S2. Baseline characteristics of publishing residents stratified by any author publication reported per resident (n=227).

Characteristic	Residents (n=227)	Any Author Publication		
		Yes (n=138)	No (n=89)	p-value
**Gender** – n(%)				
Male	132 (58.2)	77 (58.3)	55 (41.7)	0.371
Female	95 (41.9)	61 (64.2)	34 (35.8)	
**Cohort** – n(%)				
2005-2007	41 (18.1)	22 (53.7)	19 (46.3)	0.346
2008-2010	62 (27.3)	35 (56.5)	27 (43.6)	
2011-2013	65 (28.6)	45 (69.2)	20 (30.8)	
2014-2016	59 (26.0)	36 (61.0)	23 (39.0)	
**Resident Year** – mean ± SD (95% CI)	2.85 ± 2.51 (2.52, 3.18)	4.69 ± 1.29 (4.47, 4.91)	0.00	**< 0.001**
**Institution Size** – n(%)				
Small	25 (11.0)	11 (44.0)	14 (56.0)	0.123
Medium	118 (52.0)	71 (60.2)	47 (39.8)	
Large	84 (37.0)	56 (66.7)	28 (33.3)	

CI = Confidence interval; P-values < 0.05 shown in **bold**.
